# In-Situ Purification of Non-Ribosomal Peptide Synthetases Assembly Line for Structural and Biochemical Studies

**DOI:** 10.3390/ijms26041750

**Published:** 2025-02-19

**Authors:** Wei Cao, Shyue Leh Chen, Suen Kit Wu, Jialiang Wang, Zixin Deng, Jingdan Liang, Zhijun Wang

**Affiliations:** State Key Laboratory of Microbial Metabolism, Joint International Research Laboratory of Metabolic & Developmental Sciences, School of Life Sciences & Biotechnology, Shanghai Jiao Tong University, Shanghai 200240, China; davycao@sjtu.edu.cn (W.C.); chen_0429@sjtu.edu.cn (S.L.C.); skwu.0925@sjtu.edu.cn (S.K.W.); wangjialiang@sjtu.edu.cn (J.W.); zxdeng@sjtu.edu.cn (Z.D.); jdliang@sjtu.edu.cn (J.L.)

**Keywords:** NRPS, in-situ purification, affinity tag, MbtH-like protein

## Abstract

Nonribosomal peptide synthetases (NRPS) are essential for the biosynthesis of therapeutically valuable molecules, including antibiotics, immunosuppressants, and anticancer agents. The assembly-line mechanism of NRPS offers significant potential for engineering novel natural products through reprogramming. However, the challenging purification of NRPS proteins has impeded the investigation of their assembly and catalytic mechanisms. In this study, we employed homologous recombination to insert a purification tag at the C-terminus of the NRPS gene within the chromosome. This genetic modification enabled efficient purification of NRPS proteins from the tagged mutant strain using a one-step affinity chromatography approach. Additionally, we discovered that MbtH-like proteins (MLPs) form stable complexes with all pyoverdine (PVD) NRPS subunits, allowing for the purification of the entire NRPS assembly line via tagged MLP. Negative stain electron microscopy analysis revealed that the purified PVD NRPS proteins exist as dynamically linear monomers. Our in-situ tag-based purification method enhances NRPS research in both biochemical and structural biology, providing a robust platform for further investigations into NRPS mechanisms and applications.

## 1. Introduction

Nonribosomal peptide synthetases (NRPS) play a pivotal role in the production of various therapeutically significant molecules, including antibiotics such as penicillin and daptomycin, immunosuppressants like cyclosporine A, and anticancer drugs such as bleomycin [1,2,3,4]. NRPS utilize a remarkable modular synthesis mechanism, where distinct domains within each module catalyze specific steps to incorporate particular amino acid residues into the growing peptide chain. These modules are sequentially arranged into an assembly line, ensuring that each amino acid is loaded at its designated position within the final product [5,6]. This modular architecture enables NRPS to synthesize peptides with diverse structures and functionalities, demonstrating their versatility and potential for generating novel therapeutic agents [7,8].

Despite their significant role, the expression and purification of NRPS remain challenging endeavors, particularly at the assembly line level [9]. The complexity and large size of these enzymes often impede their isolation and characterization, limiting our ability to fully harness their synthetic potential. Overcoming these obstacles is crucial for advancing our understanding and utilization of NRPS in peptide drug discovery and development.

Heterologous expression of NRPS proteins is extremely difficult, primarily due to their substantial molecular weights. NRPS are giant enzymes, with a typical module for amino acid loading being approximately 110 kDa. A single NRPS subunit is usually composed of one or more modules, implying that a single subunit weighs at least 100 kDa or more [5,10]. The full NRPS assembly line typically ranges in size from millions of Daltons (MDa). For instance, the synthetase SimA, responsible for synthesizing the immunosuppressant cyclosporine A, comprises 11 modules with a total of 15,281 amino acid residues, weighing approximately 1.69 MDa [2]. Such sizes make it challenging to clone, express, and purify intact NRPS subunits using conventional methods.

Early studies attempted to purify NRPS proteins in situ, i.e., from their native expressing bacteria post-fermentation, where these proteins perform their biochemical functions [2,11]. However, purification methods relying on non-tagged techniques, such as ammonium sulfate precipitation, PEG precipitation, ion exchange chromatography, and gel filtration chromatography, often yield very low purification efficiencies. Large amounts of bacterial biomass may be required to purify only small quantities of protein, and the purity of the protein may not be sufficient for in vitro biochemical and structural studies.

In this study, we first employed a gene recombination method to knock in purification tags at the C-termini of the NRPS subunits within a pyoverdine biosynthetic gene cluster, which can be activated to synthesize pyoverdine (PVD). We successfully purified the tagged NRPS proteins using affinity chromatography from these mutant strains. The tagged NRPS subunits were purified individually rather than as complexes, suggesting weak interactions among NRPS subunits. Subsequently, we knocked in purification tags at the C-termini of the MbtH-like protein (MLP), a chaperone protein of NRPS A domain. Affinity chromatography purification of the tagged MLP demonstrated that all NRPS subunits were co-purified with MLP, indicating that MLP forms relatively stable complexes with NRPS proteins. This approach allows for the purification of the entire NRPS assembly line. Negative-stain electron microscopy of the purified NRPS proteins revealed that each PVD NRPS subunit exists as a dynamic linear monomer, and the proteins purified through our in situ tag-based method are suitable for structural biology research. The in situ tag-based purification method for NRPS proteins developed in this study will facilitate research in the biochemical and structural biology of NRPS, providing valuable tools for further elucidating NRPS mechanisms and enhancing their application in therapeutic development.

## 2. Results

### 2.1. Knock-In of Purification Tags into NRPS Genes

**Figure 1:**
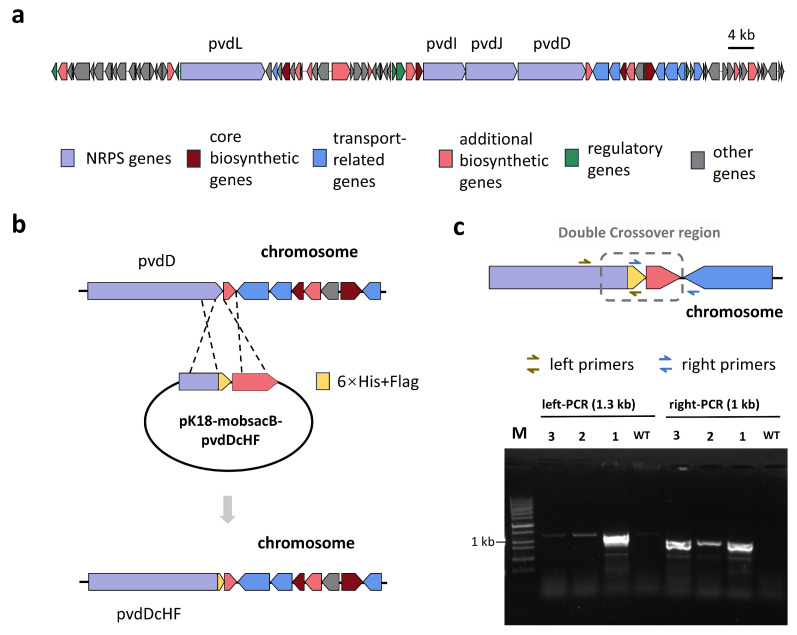
Insertion of a purification tag into the pyoverdine (PVD) biosynthetic gene cluster of *Pseudomonas putida* KT2440. (**a**) Overview of the PVD biosynthetic gene cluster in P. putida KT2440, which spans approximately 110 kilobases (kb). (**b**) Schematic representation of the method used to insert tandem purification tags, utilizing a suicide plasmid-based approach. (**c**) PCR screening workflow for verifying correct and scarless tag insertion via double-crossover events (upper panel) and representative PCR results demonstrating successful tag knock-in (lower panel).

We selected the PVD synthesis gene cluster from *Pseudomonas putida* KT2440 (Figure 1a) for tag knock-in and subsequent in situ protein purification experiments for two primary reasons: (1) *Pseudomonas putida* KT2440 is a widely used engineering bacterium with a well-established genetic manipulation system [12]; (2) PVD synthesis is regulated by iron concentration [13], allowing us to control the expression of PVD NRPS proteins by adjusting the iron levels in the culture medium. We confirmed the expression of PVD NRPS proteins under iron-deficient conditions using SDS-PAGE and subsequently established a procedure to stably activate their expression.

The incorporation of the purification tag was achieved using a well-established suicide plasmid system [14] (Figure 1b). The correct double crossover events were screened through two sets of PCRs, with primers for each set designed on the purification tag and target regions outside the left and right homology arms, respectively (Figure 1c). Due to the potential for spontaneous mutational inactivation present in a certain proportion of the SacB gene used for counter-selection [15], it was essential to verify correct double-crossover events by confirming the amplification of bands with the expected molecular weights using both left and right validation primers. Additionally, these double-crossover events were subjected to sequencing validation to ensure the scarless integration of the purification tag. We introduced only a single set of tandem affinity tags into the chromosome of each mutant strain. Utilizing the aforementioned method, we successfully constructed four mutant strains, each harboring a tandem affinity tag combination—6 × His + Flag (cHF)—at the C-terminus of different PVD NRPS proteins.

### 2.2. PVD NRPS Subunits Purified Individually Rather Than as a Complex

**Figure 2:**
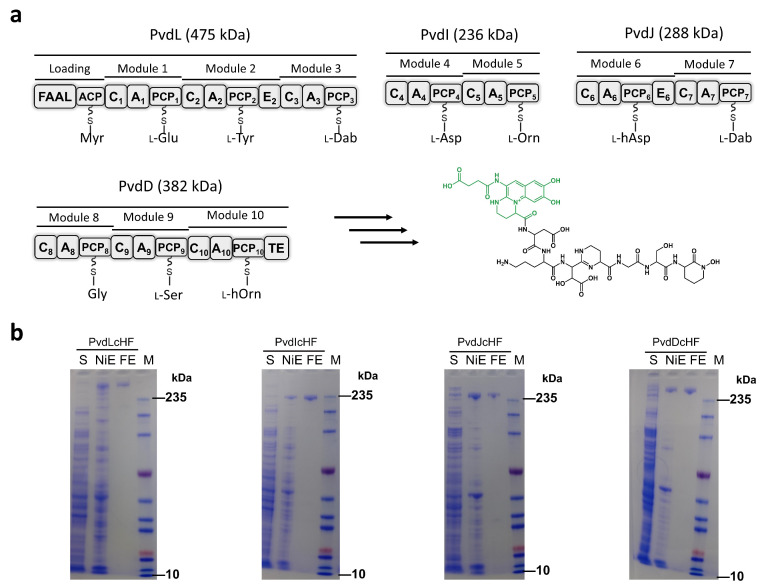
In-situ purification of the tagged PVD NRPS Protein. (**a**) Predicted molecular weight, domain composition, and functional overview of the PVD NRPS protein. NRPS domains are labelled as follow: FAAL, fatty acyl-AMP ligase; ACP, acyl carrier protein; C, condensation; A, adenylation; PCP, peptidyl carrier protein; E, epimerization; and TE, thioesterase. The following non-standard amino acid substrate abbreviations are used: Myr, myristic acid; Dab, 2,4-diaminobutyric acid; Orn, ornithine; hAsp, β-hydroxy-aspartic acid; hOrn, N^5^-hydroxy-ornithine. (**b**) SDS-PAGE analysis of the purified PVD NRPS protein. All samples were analyzed using the same molecular weight marker. Abbreviations: S, supernatant; NiE, elution of Ni resin; FE, elution of flag resin; M, marker.

The complete PVD NRPS assembly line composes four subunits: PvdL, PvdI, PvdJ, and PvdD [13,16] (Figure 2a). The synthesis of PVD requires interactions among these subunits to facilitate the transfer of intermediates between the subunits. Therefore, we hypothesized that tagging any one of the NRPS subunits would enable the co-purification of the entire assembly through their interactions.

Initially, all mutant strains were cultivated under iron-restricted conditions. No notable growth retardation was observed in the mutant strains compared to the wild type under these iron-deficient conditions, indicating that the purification tags inserted at the C-terminals of the PVD NRPS did not significantly affect the synthesis of PVD. After approximately 20 h of iron-restricted cultivation, cultures from the mutant strains were harvested. Following protein extraction, each mutant strain was purified using distinct affinity chromatography methods. Specifically, the cHF-tagged NRPS proteins in each mutant were purified using one-step affinity chromatography with Ni-TED resin for His-tag purification and Flag-M2 resin for Flag-tag purification. SDS-PAGE analysis confirmed the presence of bands corresponding to the molecular weights of the tagged proteins in all cases (Figure 2b).

However, contrary to our initial hypothesis, affinity chromatography—whether utilizing IMAC (immobilized metal affinity chromatography, His-tag) or Flag affinity purification—primarily yielded individual PVD NRPS subunits rather than stable multi-subunit complexes. This result suggests that the interactions among the PVD NRPS subunits are insufficient to maintain stable co-purification under the conditions tested. Importantly, each NRPS subunit purified via one-step Flag affinity chromatography achieved a purity level exceeding 95%, and approximately 3.3 mg of PvdL, 1.7 mg of PvdI, 4.9 mg of PvdJ, and 2.4 mg of PvdD could be purified from each mutant strain cultivated in one liter of medium, respectively.

**Figure 3:**
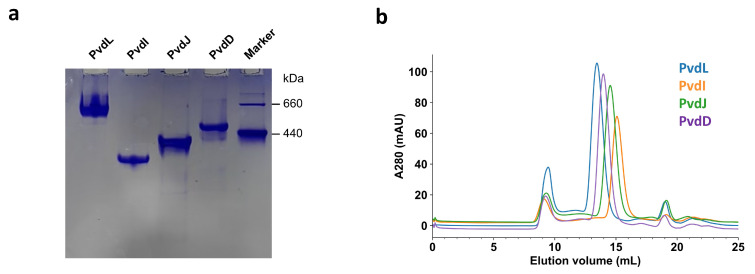
Homogeneity analysis of purified PVD NRPS protein. (**a**) Native PAGE analysis of the purified PVD NRPS protein, demonstrating its oligomeric state and purity. (**b**) Gel filtration chromatography profiling of the purified PVD NRPS protein, confirming its monodispersity and estimated molecular size. Note the peaks eluted at around 9 mL represent soluble aggregates.

To further evaluate the homogeneity and oligomeric state of the purified PVD NRPS proteins, we conducted native PAGE analysis. The results revealed that all PVD NRPS subunit bands migrated significantly below the position corresponding to dimers, indicating that the purified proteins predominantly exist as monomers (Figure 3a). Additionally, gel filtration chromatography of each purified PVD NRPS subunit displayed relatively symmetric Gaussian peaks (Figure 3b), further confirming the high homogeneity of the preparations. These findings demonstrate that the NRPS proteins purified in situ using our tag-based method are highly pure and homogeneous, making them suitable for subsequent biochemical and structural analyses.

### 2.3. One Step Purification of the NRPS Assembly Line

Previous studies have identified that many nonribosomal peptide (NRP) biosynthetic gene clusters (BGCs) include a small protein known as the MbtH-like protein (MLP), typically comprising approximately 70 amino acids [17] (∼10 kDa). MLPs are essential for the synthesis of certain NRPs [18], acting as molecular chaperones for the adenylation (A) domains of NRPS proteins, as evidenced by co-expression experiments and co-crystallization structures [19]. However, two critical questions remain: (1) Does MLP remain bound to NRPS proteins during their functional execution after assisting in their folding? and (2) Do MLP proteins associate with all NRPS subunits within a complete assembly line? To address these questions, we employed an in situ tagging purification method by knocking in a purification tag at the C-terminus of the MLP protein.

**Figure 4:**
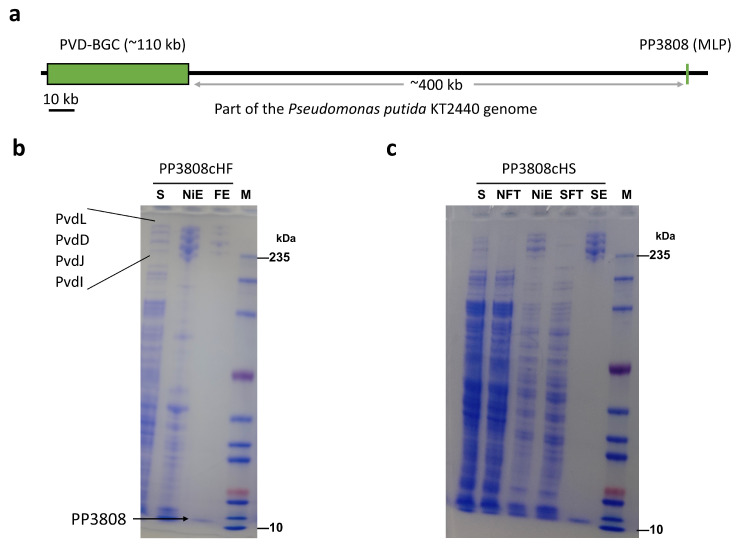
Purification of the PVD NRPS assembly line by tagging at MLP (PP3808). (**a**) Schematic diagram illustrating the location of the sole MLP gene within the *Pseudomonas putida* genome. (**b**) SDS-PAGE analysis of PP3808 protein purification with a C-terminal 6 × His + Flag tag, comparing of one-step Immobilized Metal Affinity Chromatography (IMAC) with Flag affinity chromatography. (**c**) SDS-PAGE analysis of PP3808 protein purification with a C-terminal 6 × His + Strep tag, utilizing tandem IMAC and Strep affinity chromatography. Abbreviations: S, supernatant; NiE, elution of Ni resin; FE, elution of flag resin; M, marker.; NFT, flowthrough of Ni resin; SFT, flowthrough of Streptactin resin; SE, elution of Streptactin resin.

Upon analyzing the PVD biosynthetic gene cluster (BGC) in *Pseudomonas putida* KT2440, we observed that the expected MLP gene was absent from the cluster. Instead, the only MLP gene in the genome (pp_3808) was located approximately 400 kb away (Figure 4a) and was transcribed as part of an operon with a type II thioesterase(pp_3807). In contrast, *Pseudomonas aeruginosa* PAO1 and *Pseudomonas syringae* DC3000 contain MLP genes within their respective PVD BGCs. We deduced that pp_3808 is the MLP gene involved in PVD synthesis. To test this, we utilized the scarless cloning method described previously to knock in a tandem purification tag (6 × His + Flag) at the C-terminus of the pp_3808 gene, creating the PP3808cHF mutant strain. Subsequent optimization of cultivation conditions and protein purification protocols confirmed that the tag insertion did not adversely affect the growth of *Pseudomonas putida* KT2440 under iron-deficient conditions.

Surprisingly, purification of tagged PP3808 resulted in the co-purification of all four PVD NRPS subunits (PvdL, PvdI, PvdJ, and PvdD) regardless of the affinity chromatography method used. Specifically, both one-step IMAC using Ni-TED resin and one-step Flag-M2 affinity purification yielded complexes containing all NRPS subunits alongside PP3808 (Figure 4b). Notably, one-step Flag affinity purification produced highly pure NRPS complexes. This demonstrates that tagging the MLP allows for the efficient purification of the entire NRPS assembly line, facilitating downstream biochemical reconstitution at the complete assembly line level.

However, Flag affinity chromatography is cost-prohibitive and time-consuming due to prolonged incubation periods. To address these limitations, we explored a more economical and rapid purification strategy using IMAC combined with Strep-tag affinity purification. We engineered the PP3808cHS mutant strain by inserting a 6 × His + Strep tandem tag at the C-terminus of PP3808. Following the optimized cultivation and purification protocol, SDS-PAGE analysis of the IMAC+Strep tandem affinity purification confirmed the co-purification of all PVD NRPS subunits with PP3808 (Figure 4c). This dual affinity chromatography approach enabled the recovery of approximately 10 mg of the PVD NRPS complex per liter of bacterial culture, demonstrating its efficiency and scalability.

Based on these results, we draw the following conclusions: The pp_3808 gene, located outside the PVD biosynthetic cluster, encodes the MLP involved in PVD synthesis. All NRPS subunits within the PVD assembly line exhibit strong interactions with MLP. Tagging the MLP allows for the single-step purification of all NRPS subunits, enabling the isolation of the entire NRPS assembly line.

These findings underscore the critical role of MLP in stabilizing NRPS assembly lines and provide a robust method for the purification of large, multi-subunit enzymatic complexes essential for nonribosomal peptide synthesis.

### 2.4. Linear and Dynamic PVD NRPS Subunits

The long-standing lack of structural insights into multi-modular nonribosomal peptide synthetases (NRPS) has significantly hindered their applications in synthetic biology [20,21]. The in situ purification of PVD NRPS proteins offers a valuable opportunity to elucidate the structural architecture of these complex enzymes.

**Figure 5:**
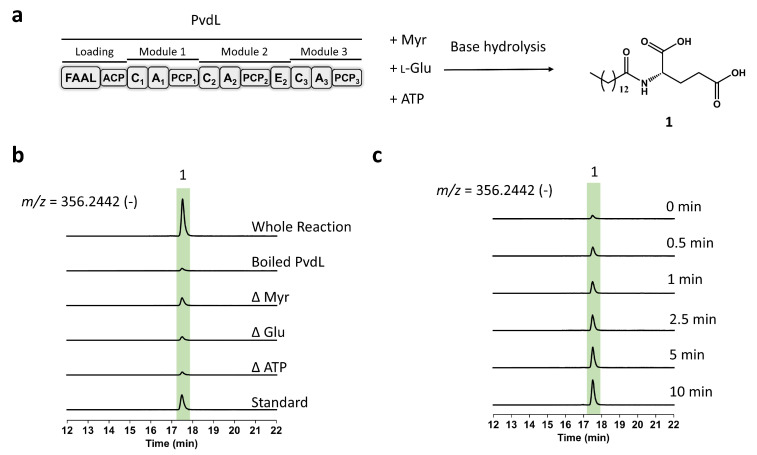
The biochemical reconstitution of PvdL in vitro. (**a**) Schematic diagram of the biochemical reconstitutions of the first two modules of PvdL. (**b**) LC/HRMS analysis of the reconstitution. (**c**) LC/HRMS analysis of the time-course experiments. The extracted ion chromatograms (EICs) obtained in negative ion mode at *m*/*z* = 356.2442 for compound **1** ([M-H]^−^). All experimental groups include three replicate controls. Abbreviation: Myr, myristic acid.

Theoretically, the proteins purified by in-situ one-step affinity chromatography should preserve high activity, making them highly suitable for biochemical and structural studies of nonribosomal peptide synthetases (NRPS). To verify this, we reconstructed the biochemical activity of the first two modules of PvdL (Figure 5a). The reconsitution experiments clearly demonstrated that compound **1** is an intermediate product generated by PvdL through the ATP-energy-dependent activation and condensation of myristic acid and glutamic acid (Figure 5b and Figure S1), which is consistent with the PVD synthesis pathway predicted based on the function of the PvdQ protein in previous articles [22,23]. The results from the substrate minus control and the boiled PvdL control suggest that the intermediate compound **1** binds to PvdL and is co-purified with it. Further comparison of the mass spectrometry of PvdL treated with and without base hydrolysis confirms this hypothesis (Figure S2). Although the reaction of the first two modules of PvdL actually involves five chemical reactions and complex protein conformational changes, time-course experiments showed that the reaction is extremely rapid, with substantial accumulation of compound **1** observable within 0.5 min (Figure 5c). Further quantitative experiments indicate that over 50% of the PvdL protein produces compound **1** within 20 min (Figure S3). These results collectively suggest that NRPS proteins purified using in-situ affinity tags retain activity.

**Figure 6:**
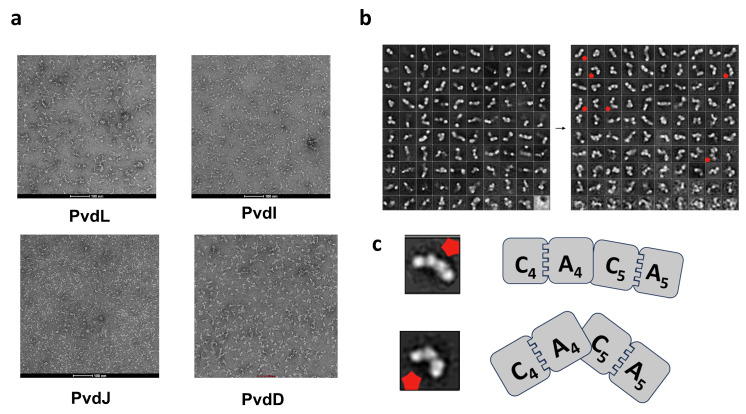
Negative stain electron microscopy analysis of the purified PVD NRPS protein. (**a**) Negative stain electron micrograph displaying the morphology of the purified PVD NRPS proteins. (**b**) 2D class averages after two rounds of classification of PvdI samples obtained from negative stain electron microscopy. (**c**) Inferred conformation of PvdI based on representative 2D classes.

To facilitate structural analysis, we purified the PVD NRPS proteins using a combination of in situ affinity chromatography and gel filtration chromatography, preparing samples suitable for negative-stain electron microscopy (EM). Upon examination with negative-stain EM, the PvdL, PvdI, PvdJ, and PvdD subunits predominantly exhibited various linear morphologies (Figure 6a). A minority of PvdL images displayed irregular spherical shapes, which, given the high purity of our samples (>95%), are likely indicative of protein aggregates rather than inherent structural features of the NRPS subunits. The predominant filamentous structures observed are thus attributed to the authentic morphology of the PVD NRPS proteins.

To gain further structural insights, we performed two-dimensional (2D) classification averaging on the EM data of PvdI, which demonstrated optimal dispersion and image contrast (Figure 6b). The 2D classification revealed a spectrum of linear conformations, ranging from nearly straight lines to zigzag patterns with varying bend angles. Notably, some class averages displayed four distinct spherical densities, with variable angles separating the first two and the last two spheres.

Analysis of existing crystal structures of single NRPS modules indicates that the condensation (C) domain and adenylation (A) domain within each module form a stable catalytic platform [24]. Additionally, studies on the two-module structures of LgrA suggest that inter-module connections in NRPS assemblies are highly dynamic [25]. Within an NRPS module, the C and A domains (∼50 kDa each) are substantially larger than the peptidyl carrier protein (PCP) domain (∼10 kDa). The PCP domain’s dynamic positioning and multiple potential binding sites likely result in it being less visible under EM, whereas the more rigid C and A domains are prominently observed.

Based on these structural considerations, we infer that the four spherical densities observed in the PvdI 2D classifications correspond to the C–A catalytic platforms of two NRPS modules (C_4_A_4_C_5_A_5_). The varying angles between these spherical structures reflect the flexible and dynamic connections between adjacent modules (Figure 5c). The varying angles between these spherical structures reflect the flexible and dynamic connections between adjacent modules.

These EM observations and subsequent analyses reveal that the PVD NRPS subunits adopt a linear and dynamic arrangement, characterized by flexible inter-module interactions. This structural plasticity is likely essential for the functional versatility of NRPS assembly lines, allowing for the efficient synthesis of diverse nonribosomal peptides.

## 3. Discussion

The successful in situ purification of the PVD NRPS assembly line presented in this study marks a significant methodological advancement in the field of NRPS research. By employing homologous recombination to insert purification tags at strategic genomic loci, we overcame longstanding challenges associated with the expression and purification of these large, multi-modular enzymes. Our findings not only elucidate the interactions within the NRPS assembly line but also provide a scalable and efficient method for isolating intact NRPS complexes, thereby facilitating future structural and biochemical investigations.

A key achievement of this study was the demonstration that tagging individual NRPS subunits (PvdL, PvdI, PvdJ, and PvdD) did not result in the co-purification of the entire assembly line, suggesting that the intrinsic interactions among these subunits are insufficiently stable under the tested purification conditions. This observation underscores the dynamic and transient nature of inter-subunit interactions within NRPS complexes, aligning with previous reports that highlight the flexible connectivity between NRPS modules necessary for their catalytic functions [25]. The high purity of individual subunits (>95%) achieved through one-step Flag affinity chromatography, however, validates the efficacy of our in situ tagging approach for isolating specific components of the NRPS machinery.

In contrast, the strategic tagging of the MbtH-like protein (MLP) PP3808 resulted in the robust co-purification of all NRPS subunits, thereby enabling the isolation of the entire PVD NRPS assembly line in a single purification step. This outcome highlights the pivotal role of MLPs as stabilizing agents within NRPS complexes, a function that extends beyond their previously established role as molecular chaperones for adenylation (A) domains [19]. Our findings suggest that MLPs maintain persistent interactions with multiple NRPS subunits during their functional execution, thereby promoting the integrity and stability of the entire assembly line. This aligns with emerging evidence that MLPs contribute to the structural organization and functional coherence of NRPS systems [17].

The successful application of dual affinity chromatography using IMAC and Strep-tag purification further enhances the practicality of our purification method, offering a cost-effective and scalable alternative to traditional Flag affinity chromatography. The ability to recover approximately 10 mg of the PVD NRPS complex per liter of culture underscores the method’s efficiency, making it a viable option for large-scale biochemical and structural studies. This methodological advancement is particularly pertinent for the field of synthetic biology, where the engineering of NRPS pathways for the production of novel peptides necessitates reliable and high-yield purification techniques.

Negative-stain electron microscopy (EM) analysis of the purified PVD NRPS proteins revealed predominantly linear and dynamic monomeric structures. The observation of various linear morphologies, including nearly straight and zigzag conformations, alongside occasional irregular spherical aggregates, provides initial insights into the structural flexibility of NRPS subunits. The presence of four distinct spherical densities in the 2D classification averages of PvdI, interpreted as C–A catalytic platforms of two NRPS modules, corroborates the notion of highly dynamic inter-module connections. This structural plasticity is likely essential for the alternating interactions required for substrate channeling and peptide elongation within the NRPS assembly line.

These EM findings are consistent with the proposed models of NRPS architecture, where the condensation (C) and adenylation (A) domains form stable catalytic platform, while the peptidyl carrier protein (PCP) domains exhibit dynamic positioning to facilitate substrate transfer [24,26]. The linear and flexible arrangement observed supports the functional versatility of NRPS systems, allowing them to adapt to the incorporation of diverse substrates and the synthesis of structurally varied peptides.

Despite these advancements, certain limitations must be acknowledged. Negative-stain EM, while valuable for assessing overall morphology and heterogeneity, does not provide the high-resolution structural details necessary to elucidate the precise arrangement of domains within the NRPS assembly line. Future studies employing cryo-electron microscopy (cryo-EM) or X-ray crystallography could offer more detailed insights into the conformational dynamics and inter-domain interactions of NRPS complexes. Additionally, while our method proves effective for the PVD NRPS system, its applicability to other NRPS pathways remains to be explored. Extending this approach to diverse NRPS assemblies will be crucial for establishing its generalizability and utility across different biosynthetic contexts.

Furthermore, the functional characterization of the purified NRPS complexes was not extensively addressed in this study. Assessing the enzymatic activity and substrate specificity of the isolated assembly line would provide critical validation of the intactness and operational integrity of the purified complexes. Such functional assays, combined with detailed structural analyses, would significantly enhance our understanding of NRPS mechanisms and inform efforts to engineer these systems for the production of novel bioactive peptides.

In conclusion, this study presents a robust and scalable in situ tag-based purification method for NRPS complexes, leveraging the stabilizing role of MbtH-like proteins to isolate intact assembly lines. The methodological advancements and initial structural insights obtained herein lay a strong foundation for future biochemical and structural investigations, potentially accelerating the engineering of NRPS systems for the sustainable production of diverse and therapeutically relevant nonribosomal peptides.

## 4. Materials and Methods

### 4.1. Materials

The *Pseudomonas putida* KT2440 strain and the pK18-mob-SacB plasmid are stored in our laboratory [12,14]. Tryptone and yeast extract were purchased from Oxoid Ltd., London, UK. The *Escherichia coli* DH5α competent cells, Phanta Max Super-Fidelity DNA polymerase, 2 × Rapid Taq Master Mix and ClonExpress Ultra One Step Cloning Kit were purchased from Nanjing Vazyme Biotech Co., Ltd. (Nanjing, China). The Ni-TED (Ni-Smart) and Streptactin resins were purchased from Smart-lifesciences Biotechnology Co., Ltd. (Changzhou, China). The Flag-m2 resin was purchased from from Sigma-Aldrich, St. Louis, MO, USA.Protein marker, kanamycin, and other molecular biology-related experimental reagents were sourced from Shanghai Sangon Biotech (Shanghai, China). The consumables related to electron microscope experiments were purchased from Beijing Zhongjing Keyi Technology Co., Ltd. (Beijing, China). The standard compound **1** (N-Myristoyl-L-glutamic acid monosodium salt) and other chemicals used in this paper were purchased from Shanghai Titan Technology Co., Ltd., Shanghai, China. Primers used in this work were synthesized by Beijing Tsingke Biotech Co., Ltd. (Beijing, China) and listed in Table S1.

### 4.2. Basic Molecular Biology Experiments

Routine experiments such as total DNA extraction, polymerase chain reaction (PCR), DNA electrophoresis, and SDS-PAGE werewere performed following protocols outlined in Molecular Cloning: A Laboratory Manual [27]. Plasmid extraction was performed using a plasmid extraction kit. The SDS-PAGE analyses were conducted using the Tris-MOPS buffer system.

### 4.3. Bioinformatics Analysis

Genomic assemblies of Pseudomonas strains producing pyoverdine (PVD) were retrieved from the National Center for Biotechnology Information (NCBI) based on literature references [28]. The identification and annotation of various PVD biosynthetic gene clusters were accomplished by submitting the genome sequences to antiSMASH [29]. To locate the MbtH-like protein (MLP) within the *Pseudomonas putida* KT2440 genome, a BLAST search was initially performed using the sequence of the representative MLP PA2412 as the query against the KT2440 proteome. This identification was further validated by conducting an HMMER search (hmmsearch) using the Profile HMM (PF03621) specific for MLPs [30].

### 4.4. Construction of the Tag Knock-In Plasmid

A method combining Gibson assembly and inverse PCR was employed to construct the tag knock in plasmid [31]. Primers were designed to amplify a gene fragment of approximately 1000 bp, encompassing 500 bp upstream and downstream of the stop codon (“TGA”) of the target NRPS gene within the KT2440 PVD synthesis cluster (primers listed in the Supplementary Table S1). These fragments included 15–18 bp homology arms homologous to the linearized pK18-mob-SacB vector. The amplified fragment was ligated into the linearized vector (pK18-mob-SacB), which was prepared by PCR amplification and DpnI digestion to remove template DNA, using the ClonExpress Ultra One Step Cloning Kit (Vazyme). The Gibson assembly product was transformed into *E. coli* DH5α cells and plated on LB agar containing kanamycin. Positive clones were screened by sequencing to obtain the pK18-mob-SacB-NPRSc plasmid (e.g., pK18-mob-SacB-pvdLc). Subsequently, reverse primers containing the purification tag sequence were employed to amplify the constructed vector immediately upstream of the “TGA” stop codon. After DpnI treatment to eliminate template DNA, the PCR product was transformed into *E. coli* DH5α. Transformants were selected and verified by sequencing to confirm the successful insertion of the tag, resulting in the tag knock-in plasmid (plasmids listed in the Supplementary Table S2).

### 4.5. Knock-In the Affinity Tag into *Pseudomonas putida* KT2440

The affinity tag was integrated into the chromosome of *P. putida* KT2440 using electroporation-based transformation, following established protocols [32]. Preparation of competent cells: Overnight-activated KT2440 was inoculated into 100 mL of fresh LB medium at 1% and cultured for approximately 4 h until the OD600 reached 0.4–0.6. The bacterial cells were then collected by centrifuging at 4000 rpm at 4 °C. The cells were resuspended in a solution of 3 mM Hepes pH 7.0, and the supernatant was removed by centrifuging again at 4000 rpm at 4 °C (this step was repeated twice). The cells were resuspended in approximately 1 mL of 3 mM Hepes pH 7.0 solution and dispensed into 100 μL aliquots, which were placed on ice for later use. Electroporation: Two microliters (∼100 ng) of the tag knock-in plasmid were added to the freshly prepared electrocompetent *P. putida* cells. The mixture was transferred to a pre-cooled 1 mm electroporation cuvette on ice. Prior to electroporation, excess water was removed from the cuvette exterior using a paper towel. Electroporation was performed using the following parameters: 1200 V/cm, 25 μF, and 200 Ω. Recovery and selection: Immediately after electroporation, cells were resuspended in 1 mL of ice-cold LB medium and transferred to a 15 mL shaking tube. The cells were incubated at 30 °C with shaking at 220 rpm for 1.5–2 h. Cultures were then plated onto LB agar containing kanamycin and incubated overnight at 30 °C to select for transformants. Counter-selection: The following day, transformants were replica-plated onto LB agar containing sucrose and onto plates without sucrose but with kanamycin. Colonies that exhibited growth inhibition on sucrose plates were considered correctly transformed. These transformants were further cultured in LB medium overnight, diluted 10^4^–10^5^ fold, and spread onto LB agar containing 10% sucrose for counter-selection. Screening and verification: Transformants were screened using two pairs of PCR primers targeting the left and right homologous arms. Successful tag knock-in was confirmed by sequencing the amplified regions. Verified mutant strains were stored in 20% glycerol at −80 °C for future use (mutant strains listed in the Supplementary Table S3).

### 4.6. Culture of Tag-Knocked-In Mutant Strains

All liquid cultures were conducted at 30 °C with shaking at 220 rpm. Day 1: At 6 p.m., the mutant strains stored at −80 °C were streaked onto LB solid plates for activation. Day 2: At 6 p.m., a single activated colony was picked and inoculated into LB broth to prepare a seed culture. Day 3: At 10 a.m., a 2% inoculum from the seed culture was transferred into nutrient-rich PTB medium (containing 20 g/L yeast extract, 12 g/L tryptone, 5 g/L NaCl, and 4 mL/L glycerol, prepared with distilled water and sterilized at 121 °C for 20 min). After 8 h of cultivation, 30 mg/L of 8-hydroxyquinoline was added, and the culture was then allowed to continue overnight. Day 4: At 9 a.m., centrifuge the bacteria at 4000 rpm and 4 °C to collect the cells. The supernatant was discarded, and the cell pellet was stored at −80 °C for preservation.

### 4.7. Purification of In Situ Expressed NRPS Protein by Affinity Chromatography

Preparation of cell lysate: Approximately 15 g of frozen bacterial cells (from a 1 L PTB culture) were thawed and resuspended in 60 mL of Buffer A (50 mM Hepes, 150 mM NaCl, 5% glycerol, pH 7.6). Cells were lysed via ultrasonication (300 W power, 2-s pulses with 3-s intervals) in an ice-water bath for a total duration of 30 min. The lysate was clarified by centrifugation at 15,000 rpm for 30 min to separate soluble proteins from insoluble debris. One-step affinity purification: For each tagged mutant strain, approximately 60 mL of the clarified lysate was incubated with 5 mL of the appropriate resin (Ni-TED for His-tag or Flag-M2 for Flag-tag) pre-equilibrated with Buffer A. The mixture was rotated at low speed at 4 °C for 1 h (extended to 4 h for Flag purification). The resin was then transferred to a chromatography column to allow unbound proteins to flow through. The column was washed with two successive batches totaling 100 mL of Buffer A to remove non-specifically bound proteins. Elution was performed using 20 mL of elution buffer: Buffer A supplemented with 200 mM imidazole for IMAC, 2.5 mM dethiobiotin for Strep-tag, or 0.1 mg/mL 3 × Flag peptide for Flag-tag purification. IMAC + Strep tandem affinity purification: The IMAC elution containing the His-tagged protein was applied to a Streptactin resin-packed chromatography column. After passing through, the resin was washed and eluted according to standard protocols, using appropriate wash and elution buffers. This dual affinity approach enabled the isolation of high-purity NRPS complexes. Post-purification analysis: SDS-PAGE was utilized to monitor the purification process and assess protein purity. The oligomeric state and homogeneity of the purified proteins were evaluated using native PAGE and size exclusion chromatography (Superose 6 Increase column with PBS buffer).

### 4.8. In Vitro Reconstitution of PvdL

In a 100 μL reaction volume, 1 μM of PvdL purified by one-step Flag affinity chromatography were combined with 200 μM myristic acid, 1 mM L-Glu, 5 mM ATP, 10 mM MgCl_2_, 1 mM TCEP, 150 mM NaCl (pH 7.6), and 50 mM HEPES (pH 7.6). Reactions were incubated at 25 °C for 1 h, then adding 10 μL of 5 M NaOH and incubating at 50 °C for 10 min, followed by neutralization with 10 μL of 6 M HCl, then adding 80 μL methanol and centrifuging at 15,000× *g* for 10 min. The supernatant was transferred into sample vials for LC/HRMS analysis. LC-HRMS analyses were performed using an Agilent 1290 Infinity LC system interfaced with a 6545 Q-TOF mass spectrometer operating in negative electrospray ionization mode. Separation was achieved on a ZORBAX SB-C18 column (5 μm particle size, 250 × 4.6 mm dimensions, supplied by Agilent Technologies, Santa Clara, CA, USA) at a flow rate maintained at 0.4 mL/min. The mobile phases comprised of A (water fortified with 0.1% formic acid) and B (methanol containing 0.1% formic acid), employing the following gradient elution profile: an initial increase from 10% to 100% B over 10 min, maintenance at 100% B for 15 min (10–22 min), a rapid decrease back to 10% B within 0.1 min (22–22.1 min), followed by a 5-min equilibration period at 10% B (22.1–25 min). Extracted ion chromatograms (EICs) were generated with a mass tolerance set at 20 ppm.

### 4.9. Negative Stain Electron Microscopy

Sample preparation: Purified PVD NRPS proteins, obtained via single-step Flag affinity chromatography, were concentrated to approximately 1 mg/mL using an Amicon® Ultra Centrifugal Filter with a 100 kDa cutoff (Millipore, Burlington, MA, USA). The concentrated sample was subjected to centrifugation at 15,000 rpm for 10 min at 4 °C to remove any remaining aggregates. A 1 mL aliquot of the supernatant was loaded onto a Superose 6 Increase column for size exclusion chromatography (SEC) using SEC buffer (50 mM Hepes, 10 mM NaCl, pH 7.6). The fraction with the highest protein concentration from the SEC elution was selected and diluted with SEC buffer to achieve a final protein concentration of 0.025 mg/mL. Grid preparation and staining: Ten microliters of the diluted PVD NRPS protein sample were drop-cast onto a hydrophilically treated copper grid with a carbon support film. The sample on the grid was fixed with uranium acetate staining to provide contrast for electron microscopy imaging. Electron microscopy: The negatively stained samples were observed and photographed using a transmission electron microscope operating at 120 keV and a magnification of 67,000×. Image processing involved particle extraction followed by two-dimensional (2D) classification and averaging using Relion 4.0 software to enhance structural features and reduce noise [33].

## Data Availability

The data presented in this study are available in this article and Supplementary Materials.

## Supplementary Materials

The following supporting information can be downloaded at: https://www.mdpi.com/article/10.3390/ijms26041750/s1.
