# Supplementary material for: In-Situ Purification of Non-Ribosomal Peptide Synthetases Assembly Line for Structural and Biochemical Studies
Source: Int J Mol Sci. 2025 Feb 19;26(4):1750. doi: 10.3390/ijms26041750 (PMC11855355; doi:10.3390/ijms26041750)
Supplement: Supplementary file 1 [file ijms-26-01750-s001.zip › ijms-3422428-supplementary.pdf]

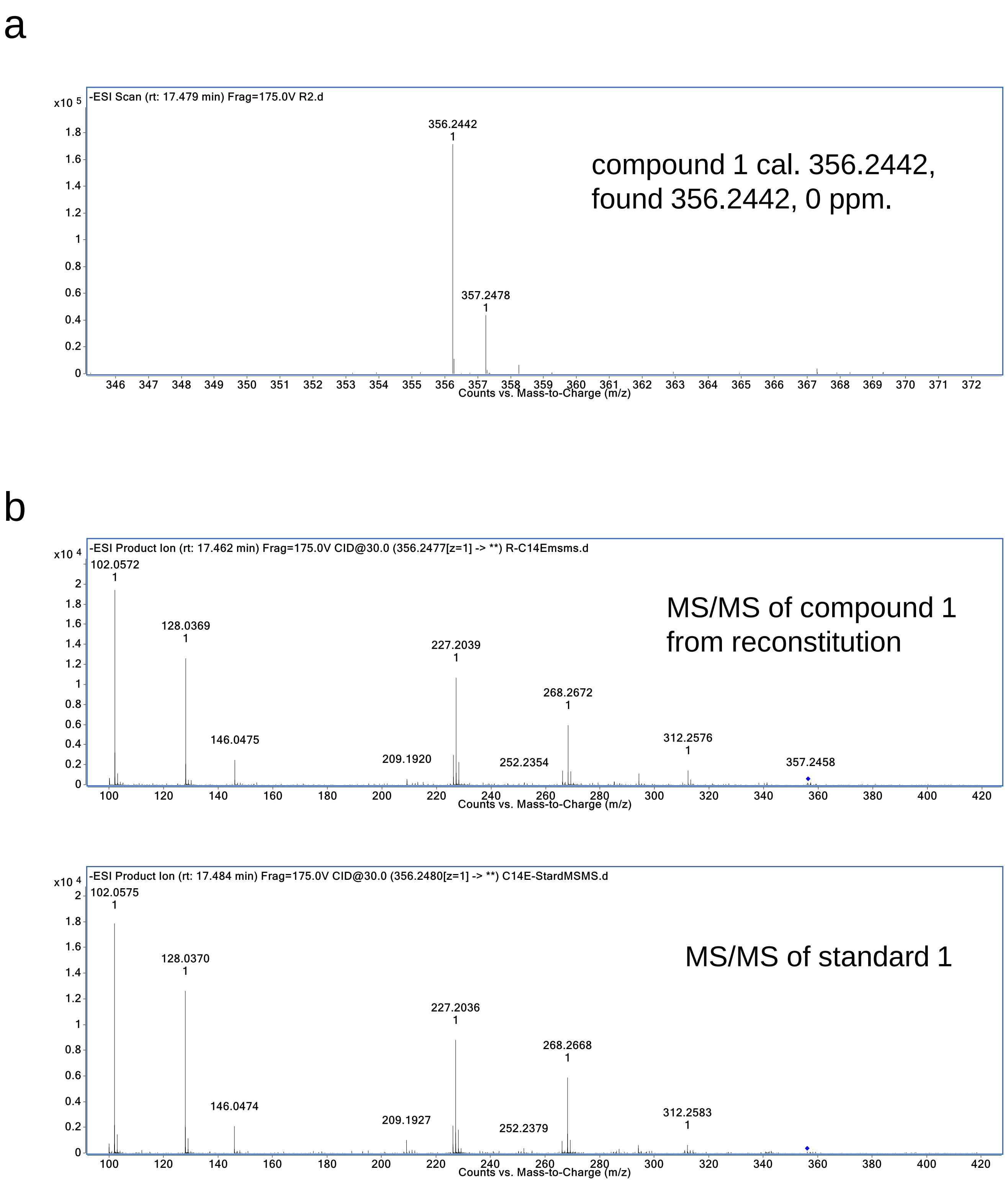

Figure S1. The mass spectrometry analysis of Compound 1. (a) High-resolution mass spectrum of Compound 1 in the reaction product. (b) Comparative MS/MS spectra: tandem mass spectrometry (MS/MS) data for compound 1 from PvdL reconstitution and standard show a maximum deviation below 20 ppm, confirming their structural equivalence.

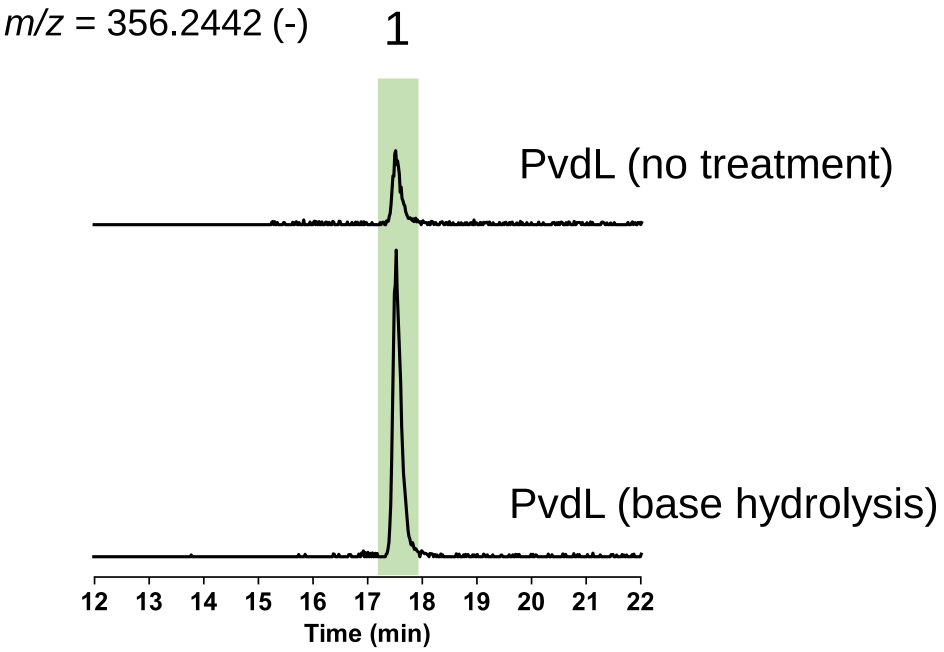

Figure S2. LC/HRMS analysis of compound 1 covalently linked to in-situ purified PvdL. The extracted ion chromatograms (EICs) obtained in negative ion mode at *m/z* = 356.2442 for compound 1 ($[M-H]^-$).

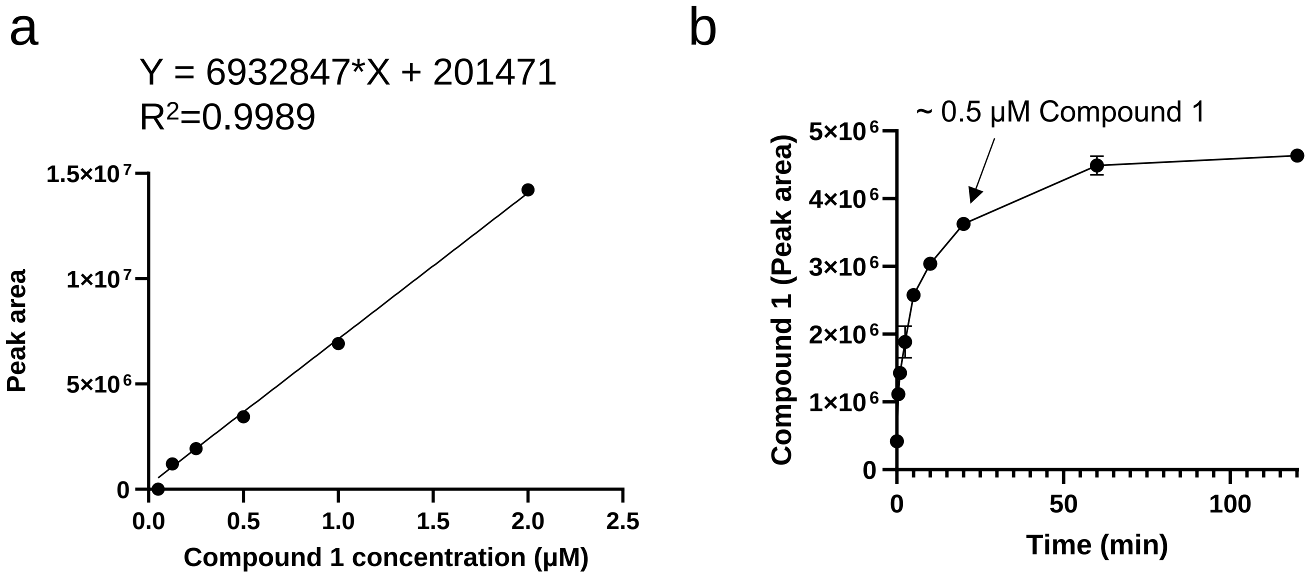

Figure S3. Quantitative analysis of biochemically reconstituted product Compound 1. (a) Standard curve and linear regression analysis for the concentration of compound 1. (b) Time course curve of Compound 1 synthesis by PvdL in vitro. The error bars represent the standard deviation. The concentration of Compound 1 was determined by substituting the peak area into the linear regression equation.

Tabel S1. The primers used in this article.

Primer name	Sequence
pK18-Linearization-SP	CTAGAGTCGACCTGCAGGCAT
pK18-Linearization-AP	CGGGTACCGAGCTCGAATT
PvdL-500TGA500-SP	CGAGCTCGGTACCCGACTGGCAGGATCCAAGTGGGC
PvdL-500TGA500-AP	GCAGGTCGACTCTAGGACGTGGCGTTCTTCCGCGTC
LcHF-SP	CCTTGTAATCATGGTGGTGATGATGGTGCAGCCCCTCCAGTTCCGCCA
LcHF-AP	ACCATGATTACAAGGATGACGACGATAAGTGATGGGCAGGGGCGCTTG
PvdI-500TGA500-SP	TCGAGCTCGGTACCCGCAGTGCGTCATAGAACGACA
PvdI-500TGA500-SP	TGCAGGTCGACTCTAGCATCCACCTGCTGGATAACA
IcHF-SP	CCTTGTAATCATGGTGGTGATGATGGTGAGCCTCAGTCAATTCATTCA
IcHF-AP	GATTACAAGGATGACGACGATAAGTGAAATGGACAAGACAACCG
PvdJ-500TGA500-SP	TCGAGCTCGGTACCCGCACCGGCAGGGCCGGCAGAT
PvdJ-500TGA500-AP	TGCAGGTCGACTCTAGGCTGGCCCATGAAGGTGTG
JcHF-SP	CCTTGTAATCATGGTGGTGATGATGGTGCTGGGTCAACTCGTCGATCTC
JcHF-AP	CACCATGATTACAAGGATGACGACGATAAGTAGCGCTCTGCGGGCCCGG
PvdD-500TGA500-SP	CGAGCTCGGTACCCGATGCAGCGGGTCGCACTCG
PvdD-500TGA500-AP	GCAGGTCGACTCTAGATGAACTGGAGCTGGCCATCG
DcHF-SP	CCTTGTAATCATGGTGGTGATGATGGTGTGAAAGTTCAATGAAGCAGG
DcHF-AP	GATTACAAGGATGACGACGATAAGTGAGCTTTCGGGGCCGCCA
PP3808-500TGA500-AP	AGGTCGACTCTAGAGCACACGGTCTTCCTGCTCAT
PP3808-500TGA500-SP	AGCTCGGTACCCGGGTTCCTTTACGCCAACCTTGC
PP3808cHF-SP	TTGTAATCATGGTGGTGATGATGGTGCTGGGCGGCAGCTTCGGCC
PP3808cHF-AP	ATGATTACAAGGATGACGACGATAAGTGACCGTGCTGAACCTGCTGTG
3808cHF2HS-AP	TGCGGGTGGCTCCACGAATCATGGTGGTGATGATGGTG
3808cHF2HS-SP	GCCACCCGCAGTTCGAAAAGTGACCGTGCTGAACCT

Tabel S2. The plasmids used in this article.

Plasmid Name	Description	Resistance
pK18-PvdLcHF	A derivative plasmid of pK18-mobsacB carrying a 1000 bp homologous gene fragment from the C-terminus of the PvdL protein fused with a C-terminal Flag tag + 6*His tag	Kan^r
pK18-PvdIcHF	A derivative plasmid of pK18-mobsacB carrying a 1000 bp homologous gene fragment from the C-terminus of the PvdI protein fused with a C-terminal Flag tag + 6*His tag	Kan^r
pK18-PvdJcHF	A derivative plasmid of pK18-mobsacB carrying a 1000 bp homologous gene fragment from the C-terminus of the PvdJ protein fused with a C-terminal Flag tag + 6*His tag	Kan^r
pK18-PvdDcHF	A derivative plasmid of pK18-mobsacB carrying a 1000 bp homologous gene fragment from the C-terminus of the PvdD protein fused with a C-terminal Flag tag + 6*His tag	Kan^r
pK18-PP3808cHF	A derivative plasmid of pK18-mobsacB carrying a 1000 bp homologous gene fragment near the PP3808 protein fused with a C-terminal Flag tag + 6*His tag	Kan^r
pK18-PP3808cHS	A derivative plasmid of pK18-mobsacB carrying a 1000 bp homologous gene fragment near the PP3808 protein fused with a C-terminal Strep tag + 6*His tag	Kan^r

Tabel S3. The mutant strain constructed in this article.

Mutant strain	Description
KT2440-PvdLcHF	The DNA coding sequence for a 6×His+Flag tandem tag was inserted before the stop codon "TGA" at the C-terminus of the pvdL gene in *Pseudomonas putida* KT2440.
KT2440-PvdIcHF	The DNA coding sequence for a 6×His+Flag tandem tag was inserted before the stop codon "TGA" at the C-terminus of the pvdI gene in *Pseudomonas putida* KT2440.
KT2440-PvdJcHF	The DNA coding sequence for a 6×His+Flag tandem tag was inserted before the stop codon "TGA" at the C-terminus of the pvdJ gene in *Pseudomonas putida* KT2440.
KT2440-PvdDcHF	The DNA coding sequence for a 6×His+Flag tandem tag was inserted before the stop codon "TGA" at the C-terminus of the pvdD gene in *Pseudomonas putida* KT2440.
KT2440-PP3808cHF	The DNA coding sequence for a 6×His+Flag tandem tag was inserted before the stop codon "TGA" at the C-terminus of the pp_3808 gene in *Pseudomonas putida* KT2440.
KT2440-PP3808cHS	The DNA coding sequence for a 6×His+Strep tandem tag was inserted before the stop codon "TGA" at the C-terminus of the pp_3808 gene in *Pseudomonas putida* KT2440.
